# Lightweight Data-Security Ontology for IoT †

**DOI:** 10.3390/s20030801

**Published:** 2020-02-01

**Authors:** Pedro Gonzalez-Gil, Juan Antonio Martinez, Antonio F. Skarmeta

**Affiliations:** 1 Dept. Ingeniería de la Información y las Comunicaciones, Facultad de Informática, Universidad de Murcia, 30100 Murcia, Spain; skarmeta@um.es; 2 Odin Solutions, Polígono Industrial Oeste C/ Perú, 5, 3∘, Oficina 12, 30820 Alcantarilla (Murcia), Spain; jamartinez@odins.es

**Keywords:** IoT, security ontolgoy, data-security, certification, regulation, provenance

## Abstract

Although current estimates depict steady growth in Internet of Things (IoT), many works portray an as yet immature technology in terms of security. Attacks using low performance devices, the application of new technologies and data analysis to infer private data, lack of development in some aspects of security offer a wide field for improvement. The advent of Semantic Technologies for IoT offers a new set of possibilities and challenges, like data markets, aggregators, processors and search engines, which rise the need for security. New regulations, such as GDPR, also call for novel approaches on data-security, covering personal data. In this work, we present DS4IoT, a data-security ontology for IoT, which covers the representation of data-security concepts with the novel approach of doing so from the perspective of data and introducing some new concepts such as regulations, certifications and provenance, to classical concepts such as access control methods and authentication mechanisms. In the process we followed ontological methodologies, as well as semantic web best practices, resulting in an ontology to serve as a common vocabulary for data annotation that not only distinguishes itself from previous works by its bottom-up approach, but covers new, current and interesting concepts of data-security, favouring implicit over explicit knowledge representation. Finally, this work is validated by proof of concept, by mapping the DS4IoT ontology to the NGSI-LD data model, in the frame of the IoTCrawler EU project.

## 1. Introduction

As world wide economies are leaning more and more on data as sources of value so does the Internet of Things (IoT) grow. Some estimates by communications companies like CISCO [1] and Ericsson [2] predict how Machine to Machine (M2M) will become one of the predominant internet traffic sources, and how mobile connections will rule over the rest.

The current status of IoT, on the other hand, is immature and, although it is growing at a steady pace, much is yet to be done in the field of security. Reports, such as Symantec’s Internet Security Threat Report [3], warn us about an increase in IoT attacks, such as the famous Mirai [4] bot-net, showed us that security, even in the case of apparently harmless devices with very reduced computing power and storage capability that did not produce, or whose produced data was not even deemed to hold any kind of strategic value in IT warfare (such as public ip cameras and DSL routers) cannot be overlooked. This is because by sheer number, those devices can be used to produce some of the strongest attacks ever recorded. Perhaps equally disturbing is the fact that modern approaches to data analysis, as well as the application of Deep Neural Network (DNN) technologies open new possibilities to infer private information by following schemes that are difficult to predict.

### 1.1. Security in IoT

Security is a pervasive concern in all Information Technologies and it is gaining even more concern in the IoT field. Power and economic constraints drive the continuous development of new devices which are continuously being deployed at an ever increasing rate. Constrained devices, which sacrifice computing power and complexity, often do so at the cost of security.

Some studies [5,6,7,8] already presented us with the many stakes, challenges and threats involved in securing the IoT, like privacy, constrained devices lacking cyrpto-power and IoT-targeted malware. IoT is spreading the attack surface of its systems by deploying hardware in uncontrolled environments, in which security analysis goes beyond the usual realms of “classical” IT and where physical damage and tampering are a very present threat, along with the use of public or shared communication channels and infrastructures, moving systems security far from what we have been used to.

**Figure 1:**
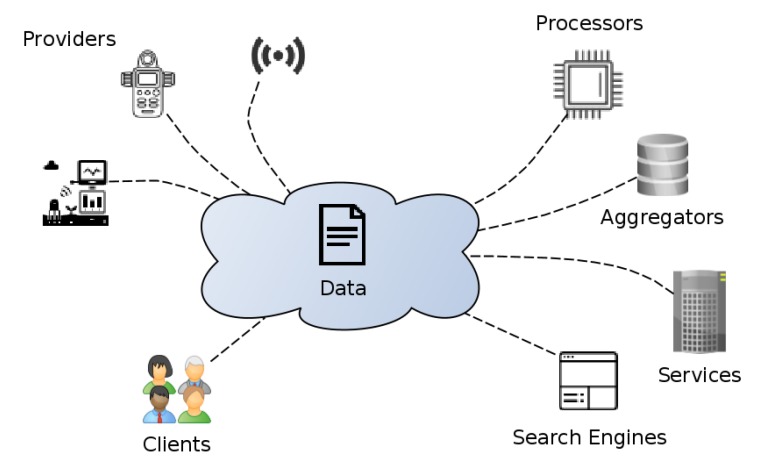
General view of different parties involved in data exchange.

It is easy to see how the mix of added complexity and the trend of leaning towards open systems (in which many different parties cooperate in an open manner, as seen in Figure 1) makes for a concerning mix. Classical security assessment relies on having a detailed picture of the description of the system, usually relying on perimeter security and/or access control to data servers, whereas IoT favours dynamic systems that change at a fast pace, moving from provider to provider and spanning many different technologies [9]. In such a scenario, it is often difficult to have a clear picture of the whole system in order to assess possible vulnerabilities.

It is also noteworthy that the way in which data flows, as well as the nature of it, also differs from the classical scenarios. Data can be consumed and processed at many different places, often difficult to trace to the origin, making it hard to track the locations it has gone through, the changes it has experienced and who has accessed it.

Some of the first problems addressed by both industry and academy have been the production of standards in the shape of protocols and frameworks, in order to homogenize and try to provide answers to many of the specific requirements imposed by IoT. As a noteworthy example of such frameworks, the FIWARE open source platform for the digital future [10] pushed the standardization of the Next Generation Service Interface (NGSI) data and communications model, around which an ecosystem of components can be deployed from a wide constellation of Enablers that provide different functionalities upon which to build platforms for IoT. Other frameworks, such as oneM2M, have also sprouted security components [11].

Finally, public and political concern has coalesced in the form of public regulations, such as the General Data Protection Regulation (GDPR), which impose a new set of difficulties and restrictions on the way in which personal data is handled, that directly affects the IoT [12].

### 1.2. Semantic Technologies in IoT

As we previously mentioned, data itself presents new challenges to the IoT scenery, namely how to structure and enrich data coming from usually simple devices. To better illustrate the problem we could imagine a temperature sensor, which regularly outputs readings. Some party interested in temperature readings would be also interested in knowing the units of measure (${}^{\circ }$C), the precision of the measurement, the intervals at which those measurements are made or when was the last time a reading was performed.

In a closed system, most of that information can be implicit to the system, already known as part of its lore, but not in the case of IoT. Further on with the problem there is the need to be able to communicate information so that every possible party can know what each of those attributes mean (think of different names for attributes in different countries). This time, though, there was an existing approach, coming from the world of Web Technologies, which already proposed a successful solution, giving birth to the Semantic Web and the spawning of many general-purpose ontologies, describing the most common knowledge about data populating the World Wide Web.

The integration of Semantic Technologies in the IoT [13], targeting world-wide services, brought with it the concept of Context, representing the merge between simple data, like a temperature reading, and the meta information that gives meaning to it, like the location of the sensor, the units of measure, the interval, the area observed by the sensor, and so forth. For that matter, many ontologies regarding IoT specific knowledge have already been created [14,15].

Some standards for IoT have already started to adopt semantics. Such is the case of the NGSI-LD [16] standard, the evolution of OMA NGSI-9/10 that has adopted JSON-LD [17], an W3C standard for linked data representation—as its data exchange format, bringing with it new possibilities such as semantic reasoning that could help to build new agents for helping with automated testing, or semantic discovery. This is particularly interesting for search engines, data markets, processors and aggregators.

### 1.3. Contribution

The focus of this work is in both semantic technology and security in the scope of IoT. In this paper we present an ontology for the description of different data security traits involved in data access and exchange in IoT systems. It represents a common vocabulary describing the practical security aspects related to data access and exchange relevant to producers, consumers and intermediaries. Its objective is to annotate data in order to facilitate its provision, access and handling, as well as to provide relevant information about regulations that may affect it, and certifications and provenance. Its purpose is to serve as a common vocabulary supporting the description of the security mechanisms associated with data and data exchange, which are strategic and crucial in different use case scenarios, such as data providers, aggregators and processors, catalogues, search engines and M2M.

In contrast to previous works, which generally offer a way of describing security from a systems perspective, this work focuses on giving a bottom-up perspective from the point of view of data itself, which serves two distinct purposes—to ease the provision, access and handling of data in the current IoT scenarios previously described, as well as giving a more practical and approachable view of security in open systems, where the security description of the whole system, as a collection of hardware, software and communication elements is often unfeasible.

The rest of this paper is structured as follows: in Section 2, the state of the art in security ontologies for IoT is covered by performing a bibliographical review. Ontology creation methodologies have also been covered, as well as some other related works that we found interesting from the perspective of this work. Section 3 describes the problem and its foundations. Section 4 describes the resulting artefact in the form of an ontology, which is later evaluated by proof of concept in Section 5. Finally, in Section 6 we share the conclusions of our work.

## 2. State of the Art

This section begins by covering some of the available ontological methodology resources followed for the development of this work, as well as a bibliographical review of the main security ontologies existing, some of which are specifically aimed at the IoT scenario. Lastly, some works that showcase the use of ontologies to perform semantic reasoning on different aspects of security, from policy expressiveness improvement, to context-aware access control that we found interesting during the development of our work, as they provide useful concepts or innovative approaches applicable to our domain.

### 2.1. Ontology Methodologies

Ontology Development 101: A Guide to Creating Your First Ontology [18], by the creators of Protègè, is one of the first go-to works on ontology creation. It describes an ontology-development process for declarative frame-based systems, as well as some of the common pitfalls and errors in which novel creators can fall.

The Mentor Methodology [19], defined as a “methodology for enterprise reference ontology development” enhances knowledge sharing among organizations, allowing its users to keep their own knowledge representations and producing a reference ontology for the subject domain. It “brings together the building and re-engineering of ontologies related to mapping competences”.

NeOn Methodology [20] offers a range of 9 scenarios, from the creation of an ontology from scratch, to several levels o re-using, re-engineering and merging of ontological resources and design patterns. Its wider audience and diverse scenarios makes it specially interesting in our case.

### 2.2. Security Ontologies

Many security ontologies have already been developed for different contexts. The most relevant ones found during the research phase of this work are listed below. They have been selected as they are of interest to some of the application domains of IoT, are directly based on IoT, or are used as the base for other ontologies of interest for this paper. To the best of our efforts, this list is a comprehensive summary of all the relevant works related to the domain of this paper, up to date as of December 2019.

Denker et al. [21,22] more than a decade ago, in the frame of Semantic Web Services, described the DAML family of ontologies, covering many security aspects. Authentication, Authorization, AccessControl, DataIntegrity, KeyDistribution and Policy are some of the concepts modelled in great detail. This work, nevertheless, present drawbacks when applied to the IoT scenario, namely: some of the concepts described are outdated or not applicable to IoT, while more recent concepts have not been added to the ontology.

Kim et al. [23] aggregate a set of related ontologies under the name Security Ontology for Annotating Resources, improving and making them extensible by redefining concepts for added expressiveness. One of the referred ontologies it tries to improve upon is the DAML ontology which, they state, only focuses on annotating web services, whereas they focus on a more general “resource annotation”.

In “Formalizing Information Security Knowledge” [24] Fenz and Ekelhart describe a general Security Ontology, providing the ontological structure for the domain of information security, further enriched with concrete knowledge of the considered organization. The resulting ontology, which contains 500 concepts and 600 formal restrictions, organized in five sub-ontologies, is claimed to support a broad range of information security risk management approaches. Similar to what we will see in following related works, the vocabulary contains terms like Assets, Threats, Vulnerabilities, Attacks and Countermeasures, focusing on a general security description of the system that could be used as input in security assessment processes.

Herzog et al. [25] present a publicly available, OWL-based ontology of information security which models assets, threats, vulnerabilities, countermeasures and their relations. The ontology can be used as a general vocabulary and extensible dictionary of the domain of information security

Gyrard et al. [26] present STACanother security ontology, this time in the context of the ETSI M2M model; building a security knowledge base (ontology, dataset and rules) to help designers secure M2M applications during the design phase. Again, it provides a general systems overview of security, this time focused on specific IoT related technologies, describing Assets, Threats and Security Mechanisms among others.

Mozzaquatro et al. [27] present the IoT Security Ontology (IoTSec), gathering and harmonizing several related ontologies (one of which is STAC). This ontology represents knowledge about security in a similar manner as the previous work, providing an extensible and ample data-set (or catalogue of knowledge), and an expressive semantic to represent the security related traits. It aims at being the reference ontology for security in IoT, incorporating most of the aforementioned ontologies, homogenizing concepts across them.

De Franco et al. [28] present SecAOnto: an ontology that formalizes knowledge on security assessment, focusing on its aspects and particularities, addressing the relationship between information security and software assessment. Again, it is built on top of STAC and it aims at supporting methods based on rigorous assessment criteria.

Tao et al. [29] present an ontology-based security service framework supporting security and privacy preservation in interactions, by using their ontology of Security that defines a common security vocabulary shared by service providers and customers, and Semantic Web Reasoning Language (SWRL)-based reasoning. This ontology allows for explicit description of the security elements that take part in communications among devices, focusing on the integrity and confidentiality properties of information security by describing concepts such as Digital Signature, Encryption, and SecurityToken, related to data protection and access control.

Choi et al. model a security context ontology [30], on which they base a Power IoT-Cloud security service framework for its use in power IoT-Cloud environments. Using various ontological reasoning technologies they are able to respond to security intrusions intelligently. One more time, the modelled ontology represents the different threats, attacks and responses in a way closely matched to the domain problem of power metering, representing some simple concepts like if the user has password of if there is some access control to a network.

Reference [31] introduced an IoT Security Evaluation Ontology (IoTSecEv) based on IoTSec and STAC, aimed at describing security concepts of interest for different observers, by which the security of an IoT system could be evaluated, enabling the generation of personalized rankings by resource security in IoT aggregators.

Arruda et al. [32] present IoT-Privontology, as a lightweight privacy layer that builds upon IoT concepts expressed in other ontologies. It makes possible to describe policies and requirements related to privacy in IoT, allowing for policy evaluation using ontological approaches. Although it doesn’t cover aspects such as authentication or identification, it does cover some of the access control topics of interest in this work, specifically those related with policy-based access control, as well as some concepts of data protection and accounting.

### 2.3. Other Related Works

Priebe et al. [33] leverage semantic reasoning to improve attribute matching on XACML rules by presenting an extension of the XACML standard in which policies are simplified by providing an ontology-based attribute management facility.

Finin et al. [34] study the relationship between RBAC and OWL, showing two different approaches to represent RBAC model in OWL and later discuss how it can be extended to model ABAC.

In his PhD thesis, Costabello [35] presents his work “Context-Aware Access Control and Presentation of Linked Data” describing PRISSMA and Shi3ld prototypes, the second being a access control framework that leverages client context, enabling context-aware access policies for accessing linked data.

Daud et al. [36] present a delegation of access control based on semantic technologies, as an enhancement of the XACML delegation profile.

### 2.4. Summary

Out of the considered ontologies, which can be compared in Table 1, none of them are specifically designed to express security treats from the perspective of data itself, but from a top-down perspective of the different elements composing an IT system, spanning from communications, networks and computing hardware, to common services and software. Neither do they capture data-security concepts in depth, although some of them do represent concepts such as “Authentication” or “Access Control” as individuals on their ontological knowledge base, often used as grounding to perform ontological inference (for instance, listing threats to some security objective).

As for other concepts considered in this work, such as Regulations and Certifications affecting data itself, none of them are covered by the considered ontologies, and only a few of those are capable of representing concepts related to Accountability, it being just an element of the broader set defined by Provenance.

## 3. Description of the Problem

The problem we aimed to solve was the description of data-security aspects that affected IoT systems. The foundation of this problem is a rich set of use-cases from which very specific data-security description needs stemmed. Here follows an example list of some use cases and problems that motivated this work:

- How to exchange data-security requirements on a federated data scenario. Federated scenarios allow requesters to utilize different access points to retrieve data, which is sourced from a different digital location from the requesting point. This scenario makes for highly scalable systems, specially interesting in Smart Cities, among others. This federation also raises the question on how to communicate relevant data-security information between the federated elements of the system, so that access-control can be enforced.
- How to represent data security aspects of data in search engines. Data markets and search engines allow requesters to search and rank data and data sources coming from third parties which have their own security mechanisms in place. In this case, data-security annotations on the data offered by the search engine, about the original data source, can allow not only an effective way of performing filtering and ranking based on security traits of the data source, but also help the requester in the retrieval of that information from the source, specially in the case of M2M, where semantic processing is crucial.
- How to express data-security requirements from information providers. Aggregators and IoT platforms that allow data providers to register themselves on the platform, need a way to express data-security requirements in order, both, to be able to transport that information from the information provider to the platform, and between platform and any third party. Data processors and data clients would benefit information regarding data-security in those datasets in order to ease the access and handling of data.

The systematic review of General Security Ontologies offered in Section 2.2, reveals a good number of existing ontologies, some of them already aimed at IoT. It also shows that all of the referred ontologies are designed from a classical resources perspective, annotating assets: devices, services, databases, network elements, communication technologies, and so forth, generating a knowledge mesh capturing the global security structure of a system, with the general objective of performing security assessment or helping in the development of those systems.

Our initial assessment of the data-security needs for the scenarios previously mentioned, showed that we needed to annotate functional specific information regarding data-security, covering the basic aspects of security, represented by the properties of Confidentiality, Integrity and Availability, that were simply not covered by any of the previously studied ontologies.

**Figure 2:**
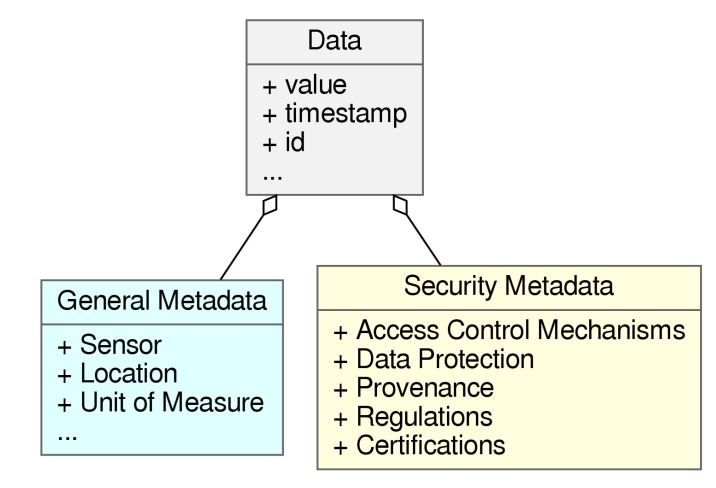
Unified Modeling Language (UML) simile of the data annotation elements related to security, identified in this work.

As depicted in Figure 2, we needed to be able to annotate information regarding at least Access Control Mechanisms, Data-Protection and Provenance. We also decided that in order to better cover the Integrity aspect of security in data, additional information could be provided in the form of Certifications, which is a field not yet developed in the scope of IoT-data-security, but that is showing interesting characteristics and academic traction [37], offering a viable candidate to help support that aspect of security. Finally, given the impact that normatives such as GDPR are having in the security aspects of data [12], we also decided to incorporate the concept of Regulation.

Finally we set a number of constraints or objectives to be met by our resulting artefact:

- Focus on data perspective, as opposed to general/systems descriptions. We aim at annotating data with security characteristics, so that interested parties can better handle, access and understand it.
- Address specific IoT problematic, such as M2M, data aggregation, personal information, end to end security and provenance.
- Favour implicit knowledge over explicit, avoiding over-describing concepts that can be extrapolated or represented by linking to external information.
- Be as lean and lightweight as possible without compromising functionality, in order to ease the learning curve and the development impact of applying it on new and current systems.
- Lends itself to be composed with other existing ontologies or used alongside with them.

### 3.1. Access Control Mechanisms

We need to provide information regarding the full process required to access some data (or part of it). IoT Frameworks, data aggregators, data catalogues and search engines can, this way, provide and use enriched links describing the minimum information required to get access to data.

Based on our prior objectives, the minimum information required to be able to access data consists on the access-control schema followed, and the authentication provider selected. Schemas such as Attribute-Based Access Control (ABAC) [38], Role-Based Access Control (RBAC) [39], Organization-Based Access Control (OrBAC), Rule-Based Access Control (RAC) and Identity-Based Access Control (IBAC) have been considered. New access-control schemas, specifically oriented towards IoT such as Distributed Capability-Based Access Control (DcApBAC) [40] that improve on ABAC’s scaling capability, require further information, such as the Capability Manager endpoint in order to obtain Capability Tokens required to successfully interact with data.

Finally, most access control mechanisms require some form of identification from the requester, which will be provided from an authentication provider (further down covered).

### 3.2. Hidden Data

Hiding data is the act of removing all traces of it from an observer, so that it cannot know if it even exists. This is different from regular access control in that when the agent requesting data is not allowed access, an error will be issued notifying the user of its inability to retrieve it, providing a clue about whether those data really exist or not, whereas hidden data will not give such clue, simply hiding from the requester the unauthorized information.

An example of this could be the displaying of personal information on a personal page. When no information is displayed, one of two things may happen: the user has not fulfilled that information, or the user has specifically prohibited it from being public so that only specific users can actually see it.

Once again, the minimum required elements to be annotated, for this approach to be possible are the requesters identification, a control point, and some kind of rules, roles or policies involved on what and when to hide the sensible data.

### 3.3. Encrypted Data

Together with the hidden data, encrypted data belong to the category of Secret Data. This time the difference lies in that the information is presented to every observer but its contents are encrypted, so that only those who hold the correct encryption key to it can actually decrypt it.

Ciphertext-Policy Attribute-Based Encryption (CP-ABE) [41] and a number of its variants are technologies of interest in the scope of IoT, relying on hiding data in plain sight by encrypting it in a manner which only the desired destinations will be able to decrypt it. This way of securing information ensures that data is stored and travels in a secure way, effectively increasing the whole system’s resiliency to a great number of threats.

Differently from regular access control mechanisms, this cryptographic schemes rely on the distribution of the crypto-keys among destinations, not needing any kind of token exchange between the data holder and the reader. In order to perform such crypto-key distribution, a Crypto Manager needs to be set in place, which additionally will require some form of identification mechanism.

### 3.4. Identification and Authentication Mechanisms

When some form of identification and authentication is required, the form in which the process is held depends on the technology selected. IoT being such a diverse scenario, it is especially the case that common authentication providers are used. By using the reference to the technology used, as well as the endpoint to perform the authentication, the most necessary and useful information required for a successful access is provided. For example, Oauth2 uses x-auth-token header, similarly the same principle can be applied to other technologies such as implementations of Verifiable Credentials [42], or other authentication mechanisms aimed at solving specific IoT constraints [43].

### 3.5. Provenance, Certification and Regulation

Provenance and certification are two challenging requirements of this work, as in the scope of IoT little work has been done so far. In that regard, the structure and properties associated with these concepts are susceptible to being updated in the future, as more alternatives are added in these fields.

For now, the most relevant information regarding certification, assuming that they will evolve in a similar manner of other existing certifications, consists of the certification reference (consisting of its URL) and some form of certificate code that would allow the interested party to consult with the certification authority the validity of the certificate, as well as specific details, like its extent or modality.

Provenance in IoT comes with its own set of challenges as well [44], although there already exist some proposals [45,46] that lean towards BlockChain usage. Again, and considering a minimalistic design, relying on implicit information, the minimum required information in order to be able to interact with provenance providers, would be to know its technology (which can be represented as its URL or web page) and endpoint where the actual queries have to be made.

Lastly, regulations are a concept tangent to security aspects. It is related to privacy, as is the case of the GDPR regulation and, as such, can be considered a rightful part of the Confidentiality attribute of data. Again, the required attributes that need to be captured by data, regarding regulations that affect it, will surely evolve, but it is safe to assume that the regulation reference (even its specific sections) can be linked by URL.

## 4. Proposal

In this section, we describe the methodology followed for the design and construction of the DS4IoT ontology, solving the problems described in Section 3, according to the constraints established there. The resulting artefact is later described in Section 4.2.

### 4.1. Methodology

For the development of the ontology, we followed the NeOn methodology, specifically that of the Scenario 1: From specification to implementation, describing the steps and processes involved in the construction of an ontology from scratch. We also considered the possibility of integrating concepts from some of the different ontologies described in Section 2, creating a reference implementation that linked to those other concepts, but finally decided against it; the reason being that although they describe in different levels of detail some of the concepts that we capture in DS4IoT, the underlying meaning is fundamentally different and could lead to inference problems and representation mismatches.

**Figure 3:**
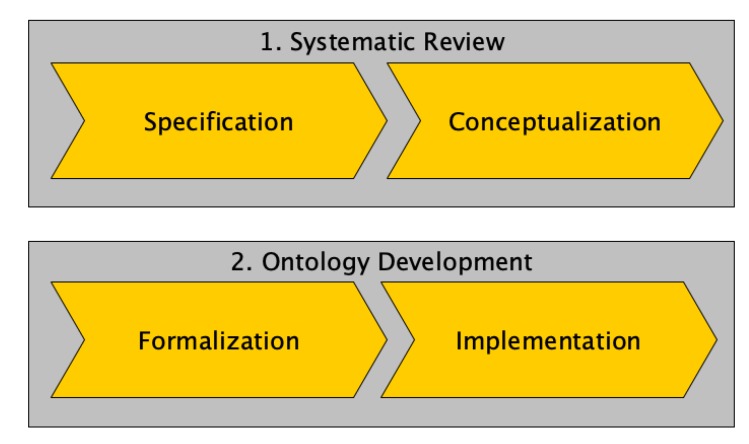
Ontology creation process.

The modelling of Data Security for IoT (DS4IoT), the overall process of which is drafted in Figure 3, begins with the problem specification, establishing the basic requirements of data annotation to solve data-security description needs on current IoT scenarios, introduced in Section 3. Next followed a study on the previous work on security, data security and its related existing ontologies scoped (where possible) to the IoT case, portrayed in Section 2. The result of this first phase was the ontology requirements specification document (ORSD) which will be used as the main guide in the next phase.

**Figure 4:**
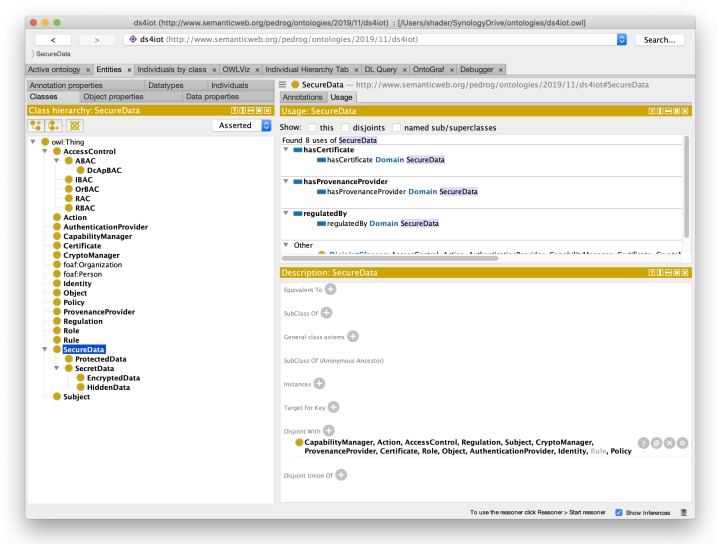
Protègè with DS4IoT loaded.

From the ORSD, we followed NeOn by carrying out the ontology formalization process, iteratively producing a vocabulary, thesaurus and concept graphs that adequately captured the minimum conceptualization of the data security traits for the IoT scope. After the formalization phase followed the ontology implementation activity in which a computable model in OWL DL language is generated. This development was performed, aided by the Protègè [47] tool, which provides a number of validation and inference helpers and visualization tools, which were very helpful in the development and validation stages. Figure 4 shows the resulting ontology and the different classes captured by it.

Together with NeOn, we also followed the Semantic Web best practices [13] commonly used on ontology creation, such as using already existing metadata definitions [48] and sharing the resulting ontology by making it publicly available on the web, as well as requesting for it to be referenced on the LOV and LOV4IoT [49] catalogues and semantic search engines. Requests to both catalogues will be issued after publication of this work. Additionally, DS4IoT was developed in the OWL DL language, using the Protègè tool during design, validation and testing phases, which is free, open source and publicly available, further easing its sharing and re-use in other works.

### 4.2. DS4IoT Ontology

**Figure 5:**
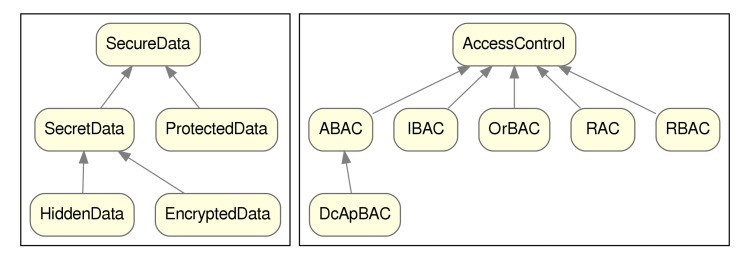
Class taxonomy. (**a**) SecureData class hierarchy. (**b**) AccessControl class hierarchy.

From the thesaurus and glossary of terms built, we modelled the different concepts related to our ontology into 25 distinct classes, 16 object properties and 3 data properties. The main two hierarchies of classes can be seen in Figure 5, depicting the families of both SecureData class and AccessControl class.

The main class of the ontology is SecureData, representing the document or fragment of data in which we are annotating data-security information. This class has a hierarchy of related sub-classes, such as SecretData, which in turn can be specialized as HiddenData or EncryptedData. None of the classes under SecureData are disjoint, meaning that any document or fragment can be tagged as any number of those classes (for example, EncryptedData and ProtectedData).

The next hierarchy of classes corresponds to the AccessControl family. This class (and its descendants) represents access control mechanisms that impose restrictions for an authenticated party on the access to some data. Some of the sub-classes are the specializations for RBAC or ABAC mechanisms (which in turn is further specialized by DcApBAC).

**Figure 6:**
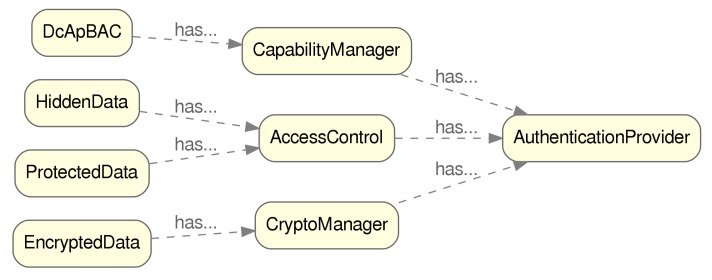
Object properties related to access control.

Figure 6 shows the main relations (data properties). Some of those links represent the relations between AccessControl, as well as the CryptoManager and CapabilityManager classes and the AuthenticationProvider class. The information regarding the endpoints related to each one of the entities associated to those classes, is held in a data property of type xsd:anyURI.

**Figure 7:**
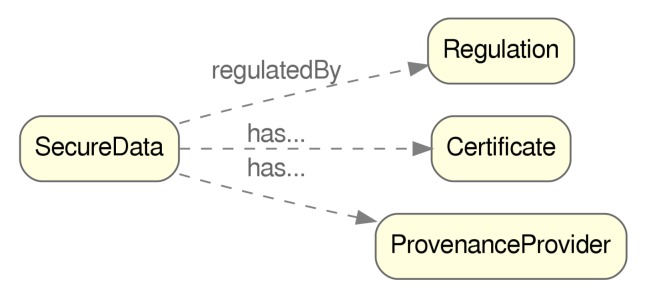
Object properties related to SecureData.

Figure 7 shows the three remaining concepts of SecureData, represented by the classes Regulation, Certificate and ProvenanceProvider, which hold data properties of type xsd:anyURI linking to the URLs of the corresponding regulations, certification authorities, and provenance providers.

Finally, some utilitarian classes to represent further information that might be used in special cases; like data provider specific data-security annotations, such as Policy *or* Organization, are also described in the ontology.

## 5. Validation and Proof of Concept

Both serving as a demo show-case of the potential of the DS4IoT ontology, as well as validation; in this section we present a proof of concept application of the DS4IoT ontology to the IoTCrawler [50] European Union (EU) funded project. This project lays the foundations for search engines and platforms for IoT data. It takes the form of a framework composing many specialized components, that share NGIS-LD as the common data-exchange interface format, and takes into consideration things like scalability of the whole system, indexing and ranking of the data registered in the platform, crawling of data from different existing domains and security, to name but a few.

**Figure 8:**
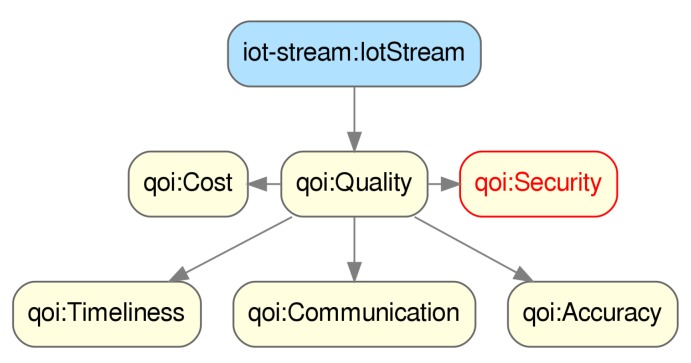
IoTCrawler ontology detail, showing the qoi:Security dimension.

In this project, security was also considered as one of the proposed dimensions of Quality of Information (QoI) as depicted in Figure 8, where we can see that one of the main concepts of IoTCrawler’s ontology is IoTStream, representing an stream of data generated by some sensor. In order to evaluate this dimension of the QoI metric for an IoT data-stream, first a description of the data-security traits associated to that stream is needed.

**Figure 9:**
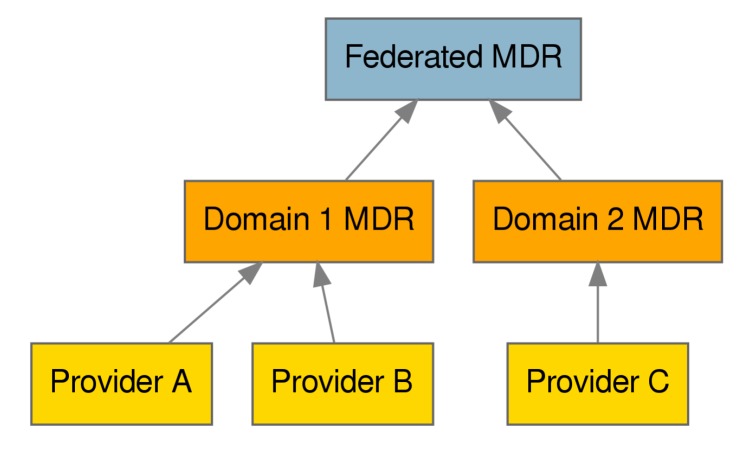
IoTCrawler federation architecture, showing regular and federated Meta-Data Repositories (MDRs).

Another aspect in which DS4IoT can benefit IoTCrawler is in the management of data-security as part of both the data-security enforcement and the access to data from different components inside IoTCrawler. Figure 9 shows how different Meta-Data Repositories (MDRs), which hold information regarding the different IoTStreams registered in the system, are structured in a hierarchical manner. This structure allows the integration of existing platforms into the IoTCrawler framework, providing at the same time, the foundation for scalability of the system. Security being a pervasive element, there is a need to communicate and represent data-security aspects across domains.

**Figure 10:**
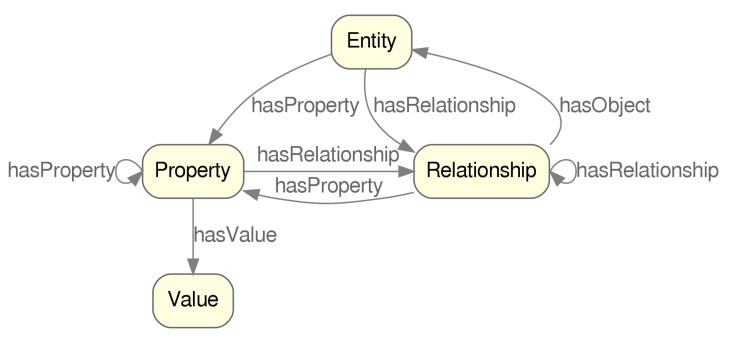
Main concepts of the NGSI-LD ontology.

The first step in order to integrate DS4IoT into IoTCrawler’s ontology, was to map DS4IoT to NGSI-LD ontology, which would later direct the data-model and representation of information in JSON-LD. In Figure 10 the main concepts of NGSI-LD are shown.

The gist of it is that information in NGSI-LD is contained in Entities which contain Relationships pointing to other entities and Properties, which contain Values. In a sense, they are very similar to objectProperties and dataProperties from OWL. Additionally, both Properties and Relationships can contain further Properties and Relationships, enriching them, adding more information. For example, given an entity which represents a sensor, a property could be the temperature reading, and properties of that reading could be the maximum and minimum values that it could take.

There are many valid alternatives for the mapping of DS4IoT to NGSI-LD, and the one we have chosen is the one that better fits its later adoption in IoTCrawler’s ontology which, as we previously said, integrates a number of ontologies, SOSA being one of them.

**Figure 11:**
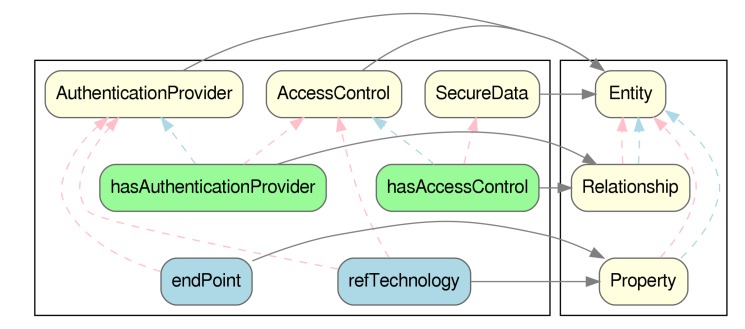
Conceptual mapping between (**a**) DS4IoT and (**b**) NGSI-LD. Classes in light yellow, object properties in light green and data properties in light blue. In dashed red and blue lines the domain and range, and in grey lines, the mapping between concepts.

Using the mapping shown in Figure 11, we can subsequently represent the access-control information required to access a sensor, represented by an entity of type IoTStream, from the IoTCrawler ontology. In Listing 1 we can see a valid NGSI-LD entity, represented in JSON-LD, which is linked via the hasAccessControl object-property (represented by an NGSI-LD Relationship), linking to another Entity.

In Listing 2 we can see the destination of the hasAccessControl relationship of the previous IoTStream. This time, an entity of type ABAC (a direct subclass of AccessControl), from the DS4IoT ontology, represents the required information required to successfully be granted access to the stream. Some more information could have been also represented here, like the contactPerson responsible for the access control of the referred IoTStream, or the CapabilityManager where the CapabilityToken had to be retrieved. In addition to the authentication provider relationship, refTechnology (mapped as a Property) gives another crucial piece of information, by linking to the URL of the FIWARE PEP-Proxy Wilma, the actual technology used to enforce access control to the stream, which is a policy-based access control system (hence the use of the class ABAC).
**Listing 1.** JSON-LD example representing an IoTStream with DS4IoT metadata.
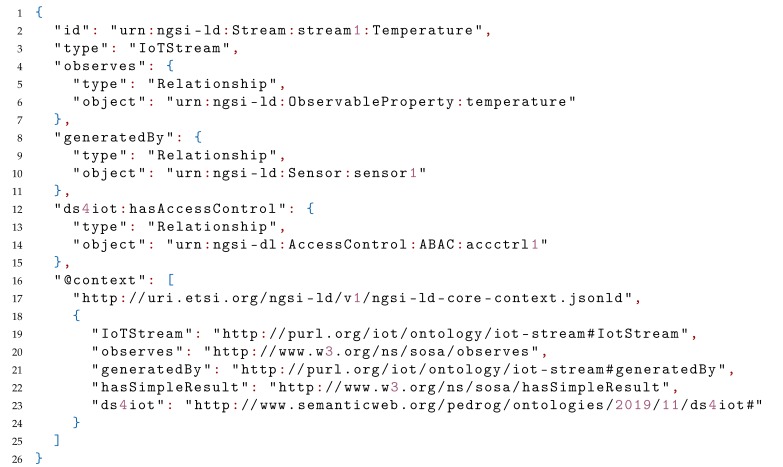

**Listing 2.** JavaScript Object Notation for Linked Data (JSON-LD) example representing an AccessControl entity.
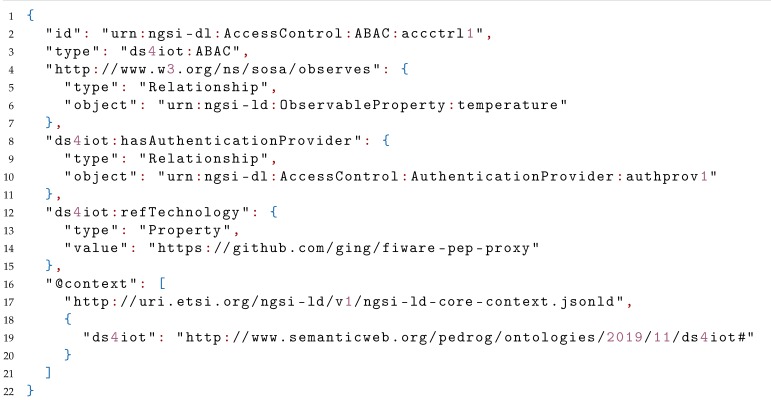


Finally, Listing 3 shows the details on the AuthenticationProvider linked by the AccessControl mechanism previously reviewed. This time, the refTechnology links to the FIWARE Keyrock IdM identity manager, based on OAuth2.0. Additionally, the ednPoint, also mapped as a Property, links to the actual endpoint where authentication has to be performed.
**Listing 3.** JSON-LD example representing an AuthenticationProvider entity.
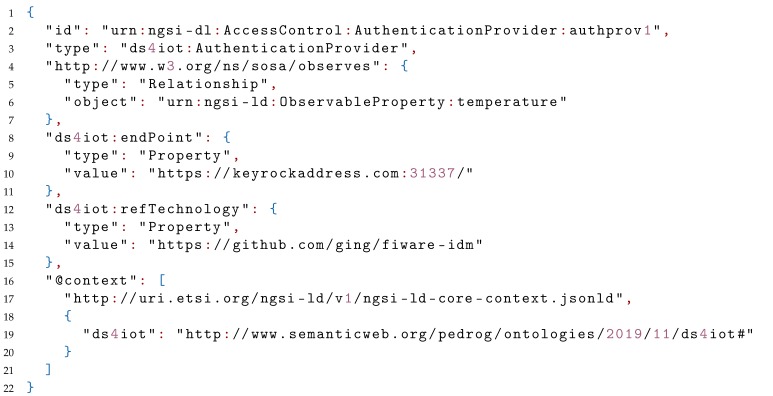


## 6. Conclusions and Future Work

Although a number of security ontologies, some of which are specifically aimed at the IoT scenery, were studied in Section 2, none are specifically aimed at, or can easily be used for, the task of annotating functional data-security aspects from the standpoint of data itself. More than that, none cover specific data-security issues related to IoT such as regulations, certifications or provenance. Those preliminary results called for a new ontology that could enable IoT frameworks, data aggregators, search engines, processors and data markets to share and consume data, by providing a vocabulary by which data-security annotations could be performed.

By following the NeOn methodology, we described and conceptualized the main concepts to represent in Section 3, to be later formalized and implemented in the resulting DS4IoT ontology described in Section 4.2. The result is a light-weight OWL DL ontology, representing current and novel concepts in the field of data-security in IoT, which favors the use of implicit over explicit knowledge, to represent the specific processes and exchanged items needed in the different traits of security that are represented. To the best of our knowledge, it is the first ontology to offer a specific vocabulary for the annotation of data-security aspects of data for IoT.

To better showcase the potential of DS4IoT ontology, as well as to empirically validate its claims, we offered a proof of concept in Section 5 in which mapping to the NGSI-LD data model was presented, as well as an adaption to the IoTCrawler ontology, covering some basic aspects of data-security that are especially relevant for the IoTCrawler EU funded project, in which a model for an NGSI-LD-driven search engine framework is currently being developed.

As a very actively developed as well as relatively recent field, new technologies, standards, frameworks and approaches at security will soon be created, and this ontology will need to be revised and updated accordingly in order to keep up and adapt to the changes to come. Especially sensible will be the concepts about regulations and certification, which have now only just begun to surface in the scope of IoT, and will surely be subject to revisions, upgrades and debate.

Additionally, other relevant aspects of data-security in the field of IoT that have been left behind in this work, such as data life-cycle, could be the subject of study for future inclusion in DS4IoT.

## Figures and Tables

**Table 1:** Comparison table of the different analysed ontologies.

Ontology	Domain	Purpose
DAML	Semantic Web Svcs.	Web Svc. security
Kim	Electronic resource	Resource annotation
SO	Information security	Risk mgmt.
Herzog	Information security	Knowledge base
STAC	IoT	Knowledge base
IoTSec	IoT	Reference ontology
Tao	Smart Home	Security service
Choi	Power IoT	Security service
IoTSecEv	IoT	Security evaluation
IoT-Priv	IoT	Privacy
